# Characterization of phosphodiesterase 2A in human malignant melanoma PMP cells

**DOI:** 10.3892/or.2013.2260

**Published:** 2013-01-31

**Authors:** HIROSHI MORITA, TAKU MURATA, KASUMI SHIMIZU, KENYA OKUMURA, MADOKA INUI, TOSHIRO TAGAWA

**Affiliations:** Department of Oral and Maxillofacial Surgery, Department of Clinical Sciences, Medical Life Science, Mie University Graduate School of Medicine, Tsu, Mie, Japan

**Keywords:** malignant melanoma, phosphodiesterase 2A, cell cycle

## Abstract

The prognosis for malignant melanoma is poor; therefore, new diagnostic methods and treatment strategies are urgently needed. Phosphodiesterase 2 (PDE2) is one of 21 phosphodiesterases, which are divided into 11 families (PDE1-PDE11). PDE2 hydrolyzes cyclic AMP (cAMP) and cyclic GMP (cGMP), and its binding to cGMP enhances the hydrolysis of cAMP. We previously reported the expression of PDE1, PDE3 and PDE5 in human malignant melanoma cells. However, the expression of PDE2 in these cells has not been investigated. Herein, we examined the expression of PDE2A and its role in human oral malignant melanoma PMP cells. Sequencing of RT-PCR products revealed that PDE2A2 was the only variant expressed in PMP cells. Four point mutations were detected; one missense mutation at nucleotide position 734 (from C to T) resulted in the substitution of threonine with isoleucine at amino acid position 214. The other three were silent mutations. An *in vitro* migration assay and a terminal deoxynucleotidyl transferase-mediated dUTP nick end-labeling assay revealed that suppressing PDE2 activity with its specific inhibitor, erythro-9-(2-hydroxy-3-nonyl)-adenine (EHNA), had no impact on cell motility or apoptosis. Furthermore, the cytotoxicity of EHNA, assessed using a trypan blue exclusion assay, was negligible. On the other hand, assessment of cell proliferation by BrdU incorporation and cell cycle analysis by flow cytometry revealed that EHNA treatment inhibited DNA synthesis and increased the percentage of G_2_/M-arrested cells. Furthermore, cyclin A mRNA expression was downregulated, while cyclin E mRNA expression was upregulated in EHNA-treated cells. Our results demonstrated that the PDE2A2 variant carrying point mutations is expressed in PMP cells and may affect cell cycle progression by modulating cyclin A expression. Thus, PDE2A2 is a possible new molecular target for the treatment of malignant melanoma.

## Introduction

The prognosis for malignant melanoma is generally poor, and effective treatment relies on early diagnosis and surgical excision with wide margins (1). Understanding the mechanisms that control the proliferation of malignant melanoma cells is crucial for developing new diagnostic methods and therapies.

Cyclic AMP (cAMP) is an intracellular second messenger. A balance between the synthesis and degradation of cAMP, catalyzed by adenylate cyclase and phosphodiesterase (PDE), respectively, regulates its intracellular concentration and thus its various physiological functions (2). The PDE superfamily of enzymes is divided into 11 families (PDE1-PDE11), and 21 genes have been identified. Approximately half of the PDE families contain more than one gene, and most of the individual genes can produce multiple mRNAs and protein sequences through alternative transcriptional start sites or alternative splicing. These isoforms show different distributions within a cell, which serve to compartmentalize specific cAMP and cyclic GMP (cGMP) signaling pathways to modulate their responses to different stimuli (2,3).

In mammals, some PDEs selectively hydrolyze cAMP or cGMP, while others hydrolyze both cAMP and cGMP. PDE2A hydrolyzes both substrates, and its binding to cGMP enhances the hydrolysis of cAMP (2,4). The PDE2A monomer is a protein of approximately 103-kDa that contains GAF-A, GAF-B and catalytic domains. PDE2A forms a homodimer, likely through the GAF-A domain. The GAF-B domain binds to cGMP, while the catalytic domain is responsible for enzymatic activity (4–6). The three splicing variants (PDE2A1, PDE2A2 and PDE2A3) of PDE2A each have a distinct N-terminal structure. These differences in the N-terminal structure appear to be responsible for the distinct subcellular localization of these variants, which determines their interactions with selective binding partners (4). Bovine PDE2A1 is soluble, while rat PDE2A2 and human PDE2A3 are membrane-bound enzymes (2,4).

We previously reported that PDE1, PDE3 and PDE5 are expressed in human malignant melanoma cells (7–9). Understanding the role of PDE2, which remains largely unknown, could be of value for developing new treatment strategies for malignant melanoma. Herein, we examined the expression of splicing variants and mutants of PDE2A in human oral malignant melanoma PMP cells. We also investigated the effect of PDE2 inhibition on cell motility, cytotoxicity, DNA synthesis, apoptosis and cell cycle progression.

## Materials and methods

### Materials

Cell culture media (RPMI-1640) and fetal bovine serum (FBS) were purchased from Invitrogen (Carlsbad, CA, USA). Phosphate-buffered saline (PBS, lacking Ca^2+^ and Mg^2+^) and erythro-9-(2-hydroxy-3-nonyl)-adenine (EHNA) were obtained from Sigma (St. Louis, MO, USA). 3-(4,5-Dimethylthiazol-2-yl)-5-(3-carboxymethoxyphenyl)-2-(4-sulfophenyl)-2H-tetrazolium (MTS) was obtained from Promega (Madison, WI, USA). The GFX PCR DNA and Gel Band Purification kit and DYEnamic ET Terminator Cycle Sequencing kit were purchased from GE Healthcare (Little Chalfont, UK). Diff-Quik stain™ was from Sysmex Co. (Kobe, Japan). Human brain total RNA was from Agilent Technologies (Santa Clara, CA, USA).

### Cell culture

Human malignant melanoma PMP cells were obtained from a 65-year-old patient with primary palatal malignant melanoma as previously described (10). Cells were maintained in RPMI-1640 medium supplemented with 5% FBS, at 37°C in a humidified 5% CO_2_ atmosphere.

### Reverse transcription-PCR (PDE2A splicing variants)

**Figure 1:**
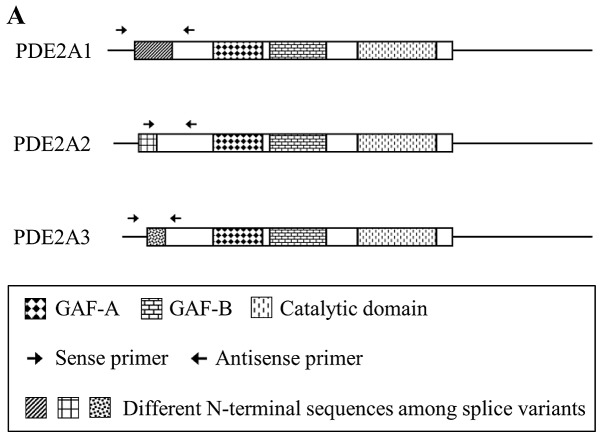

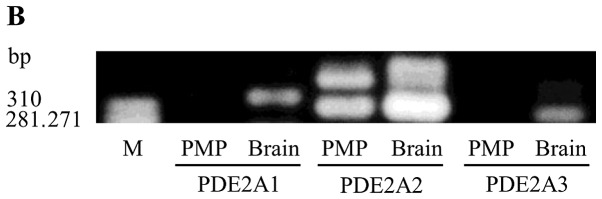

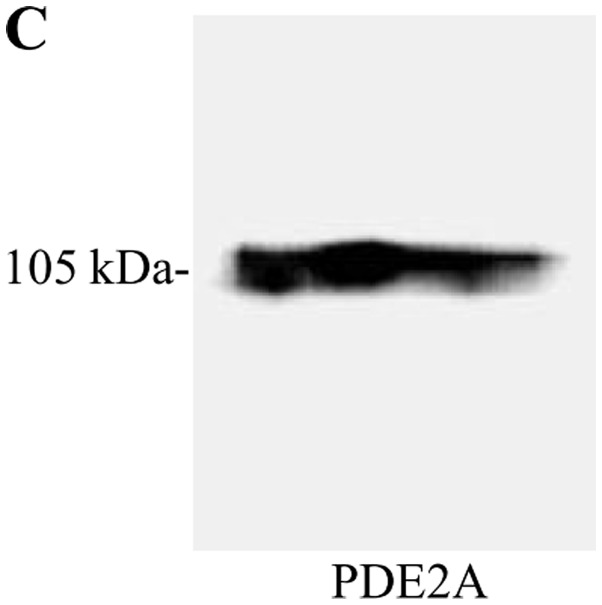
Expression of PDE2A splice variants in PMP cells. Total RNA was extracted as described in Materials and methods. cDNA was generated from total RNA and amplified by PCR using oligonucleotide primer sets based on the sequences of PDE2A splice variants. The products were separated on agarose gels and photographed after SYBR^®^ Green I staining. (A) Sense primers and antisense primers of PDE2A variants. (B) Expression of PDE2A1, PDE2A2 and PDE2A3 in PMP cells. M, molecular marker; PMP, PMP cells; Brain, human brain. (C) Western blotting of PDE2A in PMP cells. Western blotting was performed as described in Materials and methods.

PMP cells were seeded at 1×10^6^ cells/25 cm^2^ flask. After 3 days, total RNA was isolated with the QuickGene RNA Cultured Cell Kit S (Fuji Photo Film Co., Tokyo, Japan). First-strand cDNA was synthesized using total RNA with the High Capacity RNA-to-cDNA kit (Applied Biosystems, Foster City, CA, USA). PCR was performed with primer pairs specific for PDE2 splice variants (Table I, Fig. 1A). PCR amplification was carried out in a total volume of 50 μl containing PCR buffer (with 1.5 mM MgCl_2_), 200 μM dNTPs, 2.5 units HotStarTaq™ DNA polymerase (Qiagen, Hilden, Germany) and 0.5 μM sense and antisense primers. HotStarTaq DNA polymerase was activated by incubation of the reactions at 95°C for 15 min. For PDE2A1 and PDE2A2, this activation step was followed by 35 cycles of amplification (94°C for 1 min, 60°C for 1 min and 72°C for 1 min) and 72°C for 10 min. For PDE2A3, this activation step was followed by 38 cycles of amplification (94°C for 1 min, 60°C for 1 min and 72°C for 1 min) and 72°C for 10 min. Products were subjected to electrophoresis on 2.5% agarose gels and visualized by SYBR^®^ Green nucleic acid gel staining (Invitrogen).

### Western blotting

PMP cells were suspended in NuPAGE LDS sample buffer (Invitrogen). Gel electrophoresis [NuPAGE 4–12% Bis-Tris gel (Invitrogen)] was performed in an XCell SureLock Mini-Cell (Invitrogen). Following electrophoresis, proteins were transferred to iBlot Transfer stack PVDF membranes using iBlot (Invitrogen), and the membranes were blocked by incubation with PBST (0.1% Tween-20 in PBS) supplemented with 2% ECL Advance Blocking Agent (GE Healthcare) for 1 h. The blots were incubated with primary rabbit polyclonal antibody (PD2A-101AP; FabGennix, Frisco, TX, USA) overnight at 4°C and rinsed five times with PBST. Rinsed blots were incubated with horseradish peroxidase-conjugated donkey anti-rabbit IgG (GE Healthcare) for 1 h and rinsed with PBST. Immunoreactivity was detected by chemiluminescence using ECL Advance Western Blotting detection kits (GE Healthcare). The protein content was determined using BCA protein assay kits (Pierce, Rockford, IL, USA).

### Sequencing of PDE2A2

**Figure 2:**
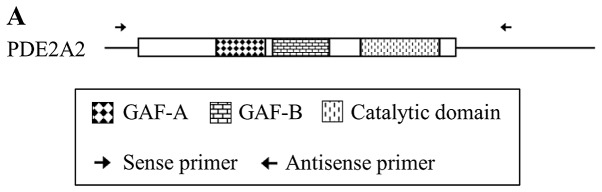

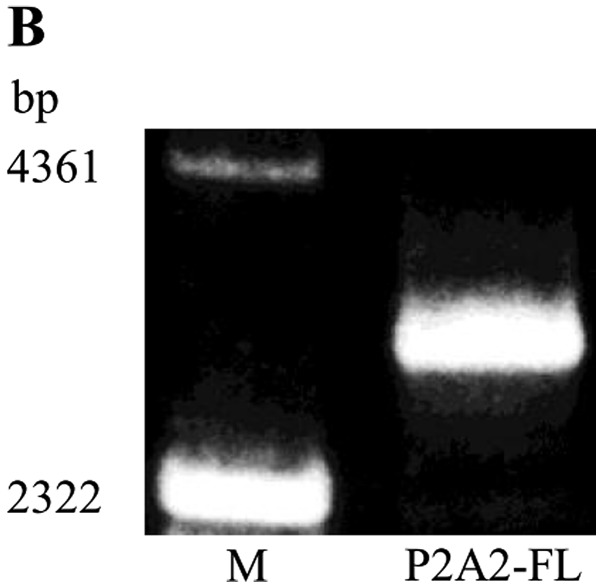

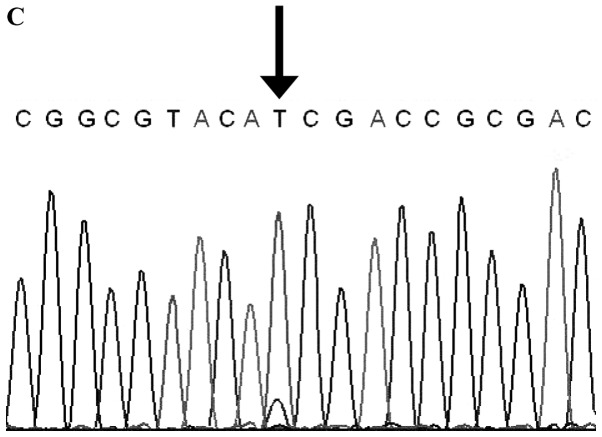
Sequencing of PDE2A2 in PMP cells. (A) Sense and antisense primers used for PDE2A2 amplification. (B) PCR product amplified by the PDE2A2 sequencing primers in PMP cells. RT-PCR was performed as described in Materials and methods. M, molecular marker; P2A2-FL, PDE2A2 full length with Fw1 and Rv1. (C) Point mutation of PDE2A2 in PMP cells. PCR products were sequenced as described in Materials and methods.

PMP cells were seeded at 1×10^6^ cells/25 cm^2^ flask. After 3 days, total RNA was isolated with QuickGene RNA Cultured Cell Kit S. First-strand cDNA was synthesized using total RNA with SuperScript^®^ II Reverse Transcriptase (Invitrogen). PCR was performed with specific primer pairs for PDE2A2 (Table II and Fig. 2A). PCR amplification was performed in a total volume of 50 μl containing PCR buffer for KOD-Plus™, 1.0 mM MgSO_4_, 200 μM dNTPs, 1 unit KOD-Plus (Toyobo, Osaka, Japan), and 0.3 μM sense and antisense primers. KOD DNA polymerase was activated by incubating the reactions at 96°C for 30 min followed by 30 cycles of amplification (94°C for 30 sec and 68°C for 3 min) and 68°C for 5 min. Products were subjected to electrophoresis on 1% agarose gels and visualized by SYBR Green nucleic acid gel staining. The PCR product generated by the PDE2A2 primers was purified using an Illustra™ GFX™ PCR DNA Gel Band Purification kit and verified by DNA sequencing.

### In vitro migration assay

PMP cells (4×10^4^ cells) in RPMI-1640 medium containing 0.1% FBS were transferred to 8-μm pore inserts (BD Biosciences), which were then placed in companion wells containing RPMI-1640 medium supplemented with 10% FBS as a chemoattractant. Following a 12-h incubation, the inserts were removed, and the non-migrating cells on the upper surface were harvested using a cotton swab. Cells on the lower surface of the membrane were fixed and stained with Diff-Quik and counted under a microscope.

### Cell growth assay

The cells were plated at a density of 400 cells/well in 96-well plates, allowed to adhere for 24 h, and then cultured in the absence or presence of different concentrations of EHNA for 3 or 5 days. MTS assays were performed using CellTiter 96^®^ Aqueous One Solution Cell Proliferation Assay (Promega), and the number of viable cells was counted.

### Trypan blue exclusion test

PMP cells (3.3×10^4^) were plated in 25 cm^2^ flasks and cultured for 24 h. Then, the cells were treated with EHNA for 3 days (50 and 100 μM) or 5 days (10, 20, 50, and 100 μM). Trypan blue stain 0.4% (Invitrogen) was added to the cell suspension, and both live and dead cells were counted under the microscope.

### BrdU cell proliferation assay

DNA synthesis inhibition was detected by a BrdU cell proliferation assay using the Amersham Cell Proliferation Biotrak ELISA System, version 2 (GE Healthcare). Cells were plated at 2000 cells/well in 96-well plates. After 24 h of culture, the cells were treated with EHNA (1, 10, 20, 50 and 100 μM) for 24 h. Cells were treated with solution A [BrdU labeling reagent plus RPMI-1640 (1:1000, v/v)] and incubated at 37°C for 2 h. After aspirating solution A, the cells were treated with fixative for 30 min, followed by solution B [blocking reagent plus redistilled water (1:100, v/v)]for 30 min. After aspirating solution B, the cells were treated with solution C [peroxidase-labeled anti-BrdU stock solution plus antibody dilution solution (1:100, v/v)] for 90 min. The cells were then washed three times with solution D [wash buffer concentrate plus redistilled water (1:10, v/v)]. The cells were treated with substrate solution for 5 min in the dark. After adding 1 M sulfuric acid to the cells, the optical density was measured in a microplate reader at 450 nm.

### Terminal deoxynucleotidyl transferase-mediated dUTP nick end-labeling assay

Apoptosis-related DNA fragmentation was detected using a terminal deoxynucleotidyl transferase-mediated dUTP nick end-labeling (TUNEL) assay using an Apop-Tag Plus Peroxidase *in situ* Apoptosis Detection kit (Millipore, Billerica, MA, USA). Cells were seeded in Lab-Tek chamber slides (Nalge Nunc International, Rochester, NY, USA) at a density of 530 cells per well. After culturing for 24 h, the cells were treated with EHNA (50 and 100 μM) for 24 h. The cells were then fixed in 1% paraformaldehyde for 10 min. The fixed cells were preserved in precooled ethanol plus acetic acid (2:1, v/v) for 5 min. Equilibration buffer was added, and the cells were incubated at 37°C for 1 h in a terminal deoxynucleotidyl transferase (TdT) enzyme solution containing deoxyuridine-5′-triphosphate-digoxigenin. After the reaction was stopped with a pre-warmed stop/wash buffer, the cells were incubated with an anti-digoxigenin antibody fragment carrying a conjugated peroxidase in a humidified chamber for 30 min at room temperature. Peroxidase activity was detected using 3,3′-diaminobenzidine as a substrate. Methyl green was applied for 20 min at room temperature for counterstaining. Total and apoptotic cell numbers were counted in 10 different fields in each well under a microscope, and the average apoptotic cell number was expressed as the percentage of the total cell number to denote the apoptotic index.

### Cell cycle analysis by flow cytometry

Cell cycle progression was analyzed using a CycleTEST™ Plus DNA Reagent kit (BD Biosciences). Cells were plated at 3.2×10^4^ cells in 25 cm^2^ flasks and cultured for 24 h. Then, cells were treated with EHNA (50 and 100 μM) for 5 days. A cell suspension was made from the 25 cm^2^ flasks. The cell suspension was centrifuged, the supernatant was aspirated, the buffer solution was added to the cells, and the cells were gently vortexed. After performing the same procedure twice, cells were counted and transferred to 15-ml plastic tubes (5×10^5^ cells/tube). The cells were centrifuged, the supernatant was aspirated, and solution A was added to the cells and incubated for 10 min, after which solution B was added to the cells and incubated for 10 min. Solution C was then added to the cells, incubated for 10 min on ice in the dark, and the cells were analyzed by flow cytometry.

### RT-PCR (CDKs and cyclins)

PMP cells (4×10^4^) were plated in 25 cm^2^ flasks and cultured for 24 h. Then, cells were treated with EHNA (50 and 100 μM) for 5 days. The total RNA of the cells was isolated using a QuickGene RNA Cultured Cell Kit S. First-strand cDNA was synthesized using total RNA with a High Capacity RNA-to-cDNA kit. PCR was performed with primer pairs specific for GAPDH, cyclins and CDKs (Table III). PCR amplification was carried out in a total volume of 50 μl containing PCR buffer (with 1.5 mM MgCl_2_), 200 μM dNTPs, 2.5 units HotStarTaq DNA polymerase (Qiagen) and 0.5 μM sense and antisense primers. HotStarTaq DNA polymerase was activated by incubation of the reactions at 95°C for 15 min. For GAPDH, this activation step was followed by 18 cycles of amplification (94°C for 1 min, 59°C for 1 min and 72°C for 1 min) with a final extension step at 72°C for 10 min. For cyclin A, this activation step was followed by 31 cycles of amplification (94°C for 1 min, 60°C for 1 min and 72°C for 1 min) and a final extension step at 72°C for 10 min. For cyclin B1, this activation step was followed by 24 cycles of amplification (94°C for 1 min, 58°C for 1 min and 72°C for 1 min) and a final extension step at 72°C for 10 min. For cyclin D1, this activation step was followed by 25 cycles of amplification (94°C for 1 min, 68°C for 1 min and 72°C for 1 min) and a final extension step at 72°C for 10 min. For cyclin E, this activation step was followed by 34 cycles of amplification (94°C for 1 min, 68°C for 1 min and 72°C for 1 min) and a final extension step at 72°C for 10 min. For CDK1, this activation step was followed by 25 cycles of amplification (94°C for 1 min, 52.5°C for 1 min and 72°C for 1 min) and a final extension step at 72°C for 10 min. For CDK2, this activation step was followed by 23 cycles of amplification (94°C for 1 min, 60°C for 1 min and 72°C for 1 min) and a final extension step at 72°C for 10 min. For CDK4, this activation step was followed by 23 cycles of amplification (94°C for 1 min, 63°C for 1 min and 72°C for 1 min) and a final extension step at 72°C for 10 min. PCR products were subjected to electrophoresis on 2.5% agarose gels and visualized by SYBR Green nucleic acid gel staining.

### Statistical analysis

All of the experiments were performed in triplicate. Differences in multiple group comparisons were analyzed using the Tukey-Kramer multiple comparisons test. Significance was defined as calculated P-values of <0.01.

## Results

### Expression of PDE2A splice variants in PMP cells

We first examined the expression of PDE2A splice variants (PDE2A1, PDE2A2 and PDE2A3) in PMP cells by RT-PCR using three specific primer pairs (Table I and Fig. 1A). PDE2A1 and PDE2A3 amplicons were not detectable, suggesting that these two splice variants were expressed at extremely low levels, or not expressed at all. Primers specific for PDE2A2 yielded a doublet by PCR (Fig. 1B), which was shown by sequence analysis to include a lower band derived from PDE2A2 (GenBank NM_001143839.3) and an upper band corresponding to the non-coding transcript variant 4 of PDE2A (GenBank NR_026572.2) with an additional 62 bp compared with PDE2A2 (data not shown). Western blot analysis showed a single band (~105 kDa) corresponding to PDE2A2 (Fig. 1C).

### Sequencing of PDE2A2 in PMP cells

We next examined the mutations in PDE2A2 expressed in PMP cells. RT-PCR using a different set of primers (Table II and Fig. 2A) yielded a 3031-bp band (Fig. 2B). Sequencing of this RT-PCR product revealed point mutations at nucleotide positions 734 (C to T), 1800 (T to C), 2268 (C to T) and 2718 (C to T). The missense mutation at position 734 resulted in the substitution of threonine with isoleucine at amino acid 214 (Fig. 2C). The other three were silent mutations.

### Effect of EHNA on the migration of PMP cells

**Figure 3:**
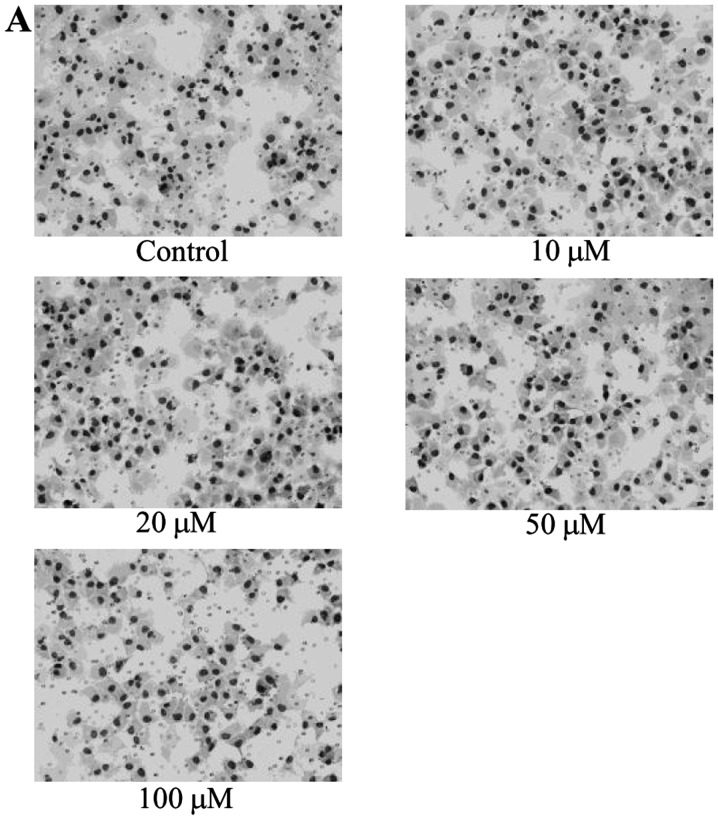

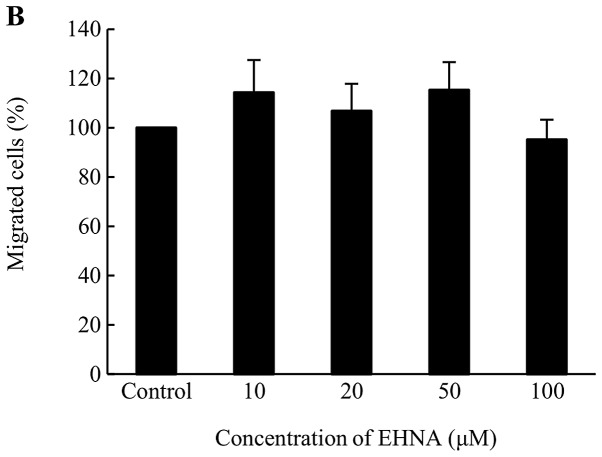
Effect of EHNA on the migration of PMP cells. The cells were transferred to inserts for migration assays after treatment with different concentrations of EHNA. The cells were stained and counted as described in Materials and methods. (A) Images of migrating cells (x200). (B) Data are expressed as the mean ± SEM of three different experiments.

Since cell motility plays an important role in metastasis, we examined whether PDE2A2 is involved in cell motility. Cells treated with 100 μM EHNA migrated in a manner similar to that of control cells (Fig. 3), indicating that inhibition of PDE2A2 did not affect the motility of PMP cells.

### Cytotoxicity of EHNA in PMP cells

**Figure 4:**
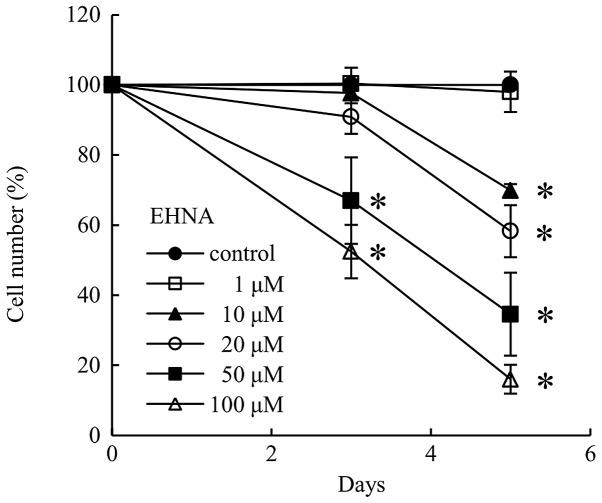
Effect of EHNA on the growth of PMP cells. Cells were plated in 96-well plates and cultured with different concentrations of EHNA. The number of cells was then counted as described in Materials and methods. Data are expressed as the mean ± SEM of three different experiments. ^*^P<0.01, significant difference from the control.

**Figure 5:**
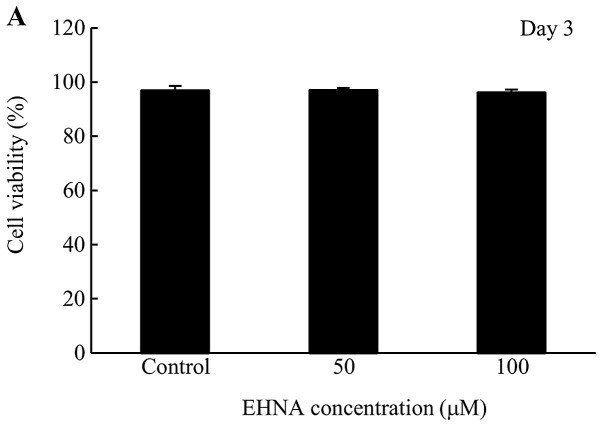

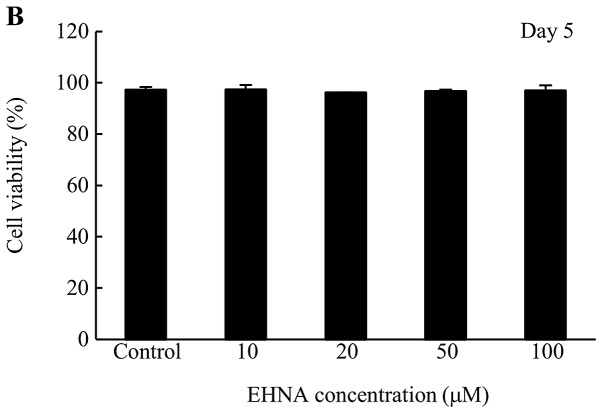
Cytotoxicity of EHNA in PMP cells. Cells were plated in 25 cm^2^ flasks and cultured with different concentrations of EHNA. Both live and dead cells were counted as described in Materials and methods. Data are expressed as the mean ± SEM of three different experiments. (A) Cell viability (%) following 3 days of EHNA treatment. (B) Cell viability (%) following 5 days of EHNA treatment.

Since EHNA-treated PMP cells grew at a slower rate than control cells (Fig. 4), we tested the cytotoxicity of EHNA using a trypan blue exclusion assay. No cytotoxicity was observed at day 3 and 5 following the addition of EHNA to PMP cells (Fig. 5).

### Effect of EHNA on DNA synthesis in PMP cells

**Figure 6:**
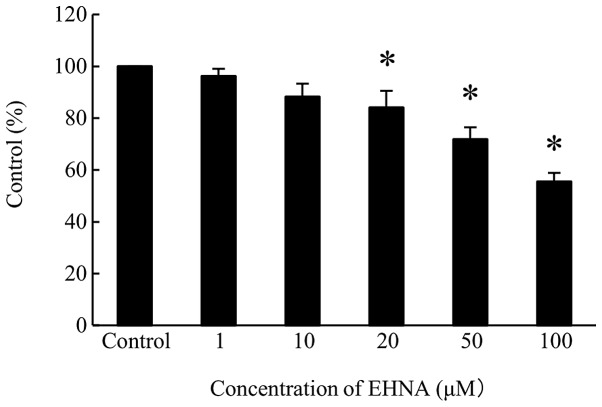
Effect of EHNA on DNA synthesis in PMP cells. DNA synthesis was detected with the BrdU cell proliferation assay as described in Materials and methods. Data are expressed as the mean ± SEM of three different experiments. ^*^P<0.01, significant difference from the control.

To examine the involvement of PDE2A in DNA synthesis, PMP cells were treated with 20, 50 and 100 μM EHNA, which decreased DNA synthesis levels to 15, 25 and 40%, respectively, of the control cell level (Fig. 6).

### Effect of EHNA on the apoptosis of PMP cells

**Figure 7:**
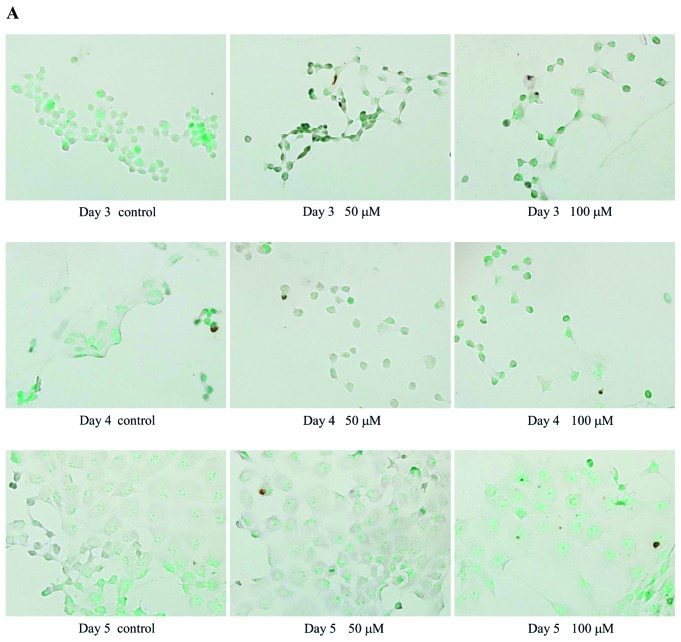

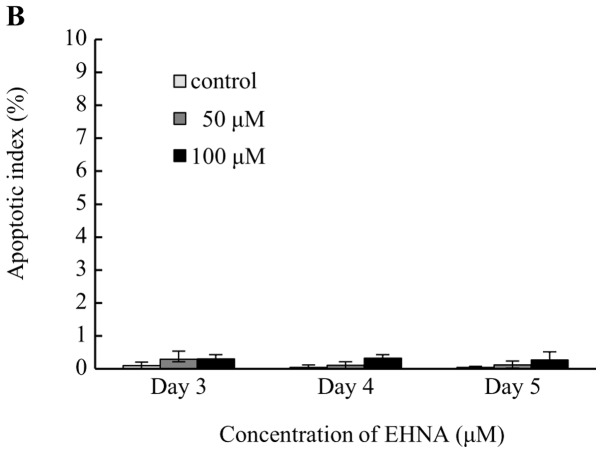
Effect of EHNA on the apoptosis of PMP cells. Apoptosis was detected by the TUNEL assay as described in Materials and methods. (A) Images of apoptotic cells (x20). (B) Data are expressed as the mean ± SEM of three different experiments.

The TUNEL assay was used to examine the effect of PDE2A inhibition on apoptosis. TUNEL-positive cells were rarely observed in EHNA-treated or control cells (Fig. 7), suggesting that PDE2A is not involved in apoptosis.

### Effect of EHNA on cell cycle progression in PMP cells

**Figure 8:**
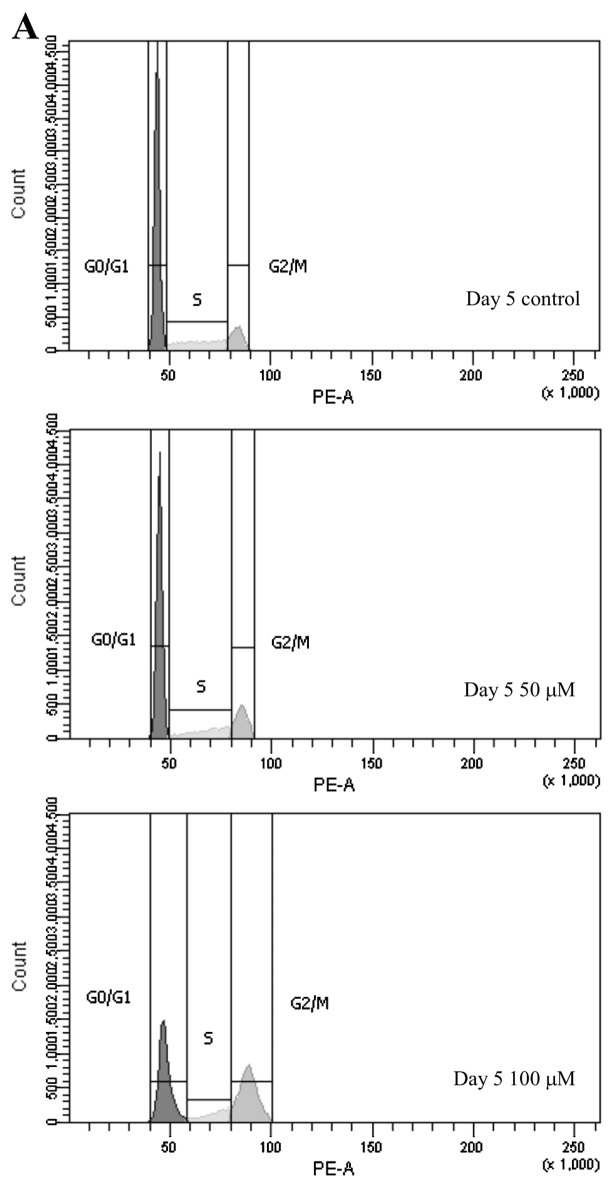

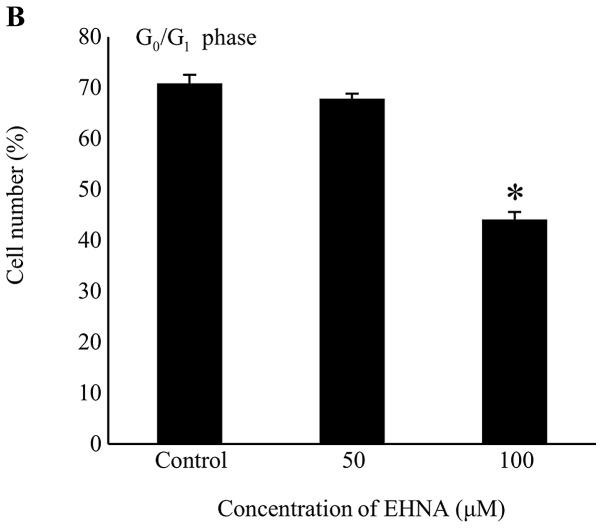

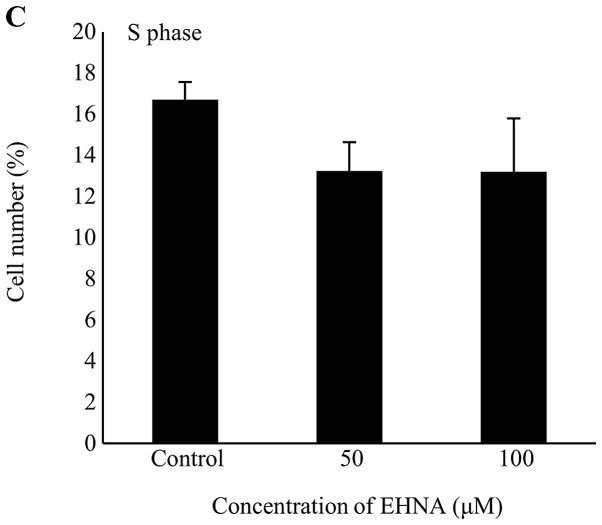

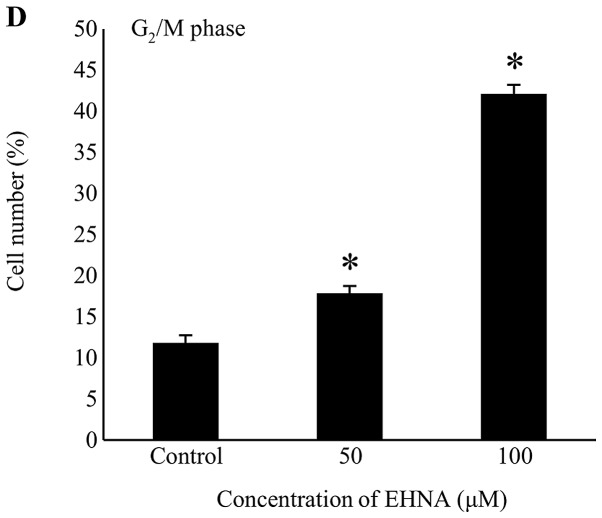
Effect of EHNA on cell cycle progression in PMP cells. Cell cycle progression was detected by flow cytometry as described in Materials and methods. (A) Histograms of the cell count following 5 days of EHNA treatment. (B) Number of cells in the G_0_/G_1_ phase following 5 days of EHNA treatment. (C) Number of cells in the S phase following 5 days of EHNA treatment. (D) Number of cells in the G_2_/M phase following 5 days of EHNA treatment. Data in B-D are expressed as the percentage of cells in each phase. Data are expressed as the mean ± SEM of three different experiments.^*^P<0.01 significant difference from the control.

Since DNA synthesis was suppressed in PMP cells treated with EHNA, we investigated the effect of PDE2 on cell cycle progression. EHNA treatment for 5 days decreased the proportion of PMP cells in the G_0_/G_1_ phase, and increased the number of cells in the G_2_/M phase (Fig. 8).

### Effect of EHNA on the expression of CDKs and cyclins in PMP cells

**Figure 9:**
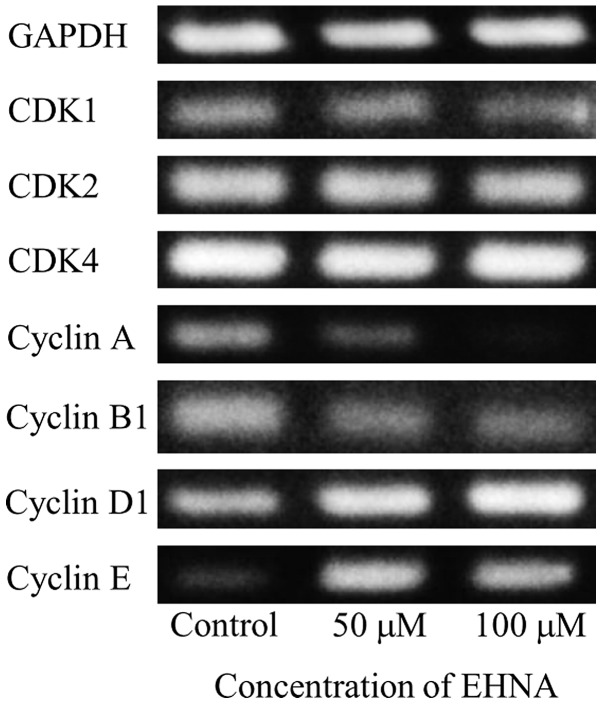
Effect of EHNA on the expression of CDKs and cyclins in PMP cells. The expression of CDKs, cyclin B and cyclin D1 was not affected by EHNA. The expression of cyclin A was inhibited, and the expression of cyclin E was stimulated by EHNA.

The effect of EHNA treatment on the expression of CDKs and cyclins was examined by RT-PCR using eight primer sets (Table III), which showed downregulation of cyclin A and upregulation of cyclin E in PMP cells treated with EHNA for 5 days (Fig. 9).

## Discussion

Malignant melanoma is associated with a poor prognosis, and there are currently no effective strategies for treating the disease in the advanced stages (1). Thus, the development of new diagnostic methods and treatments is necessary to improve prognosis. None of the conventional antitumor agents, even those used as adjunctive therapies to surgical treatment, improve the survival rates of patients with malignant melanoma, and they are not effective for controlling the disease after it metastasizes to other sites (1). Understanding the mechanisms underlying the growth, invasion and metastasis of malignant melanoma may lead to the identification of molecular targets for new diagnostic and therapeutic procedures.

PDEs hydrolyze cAMP and cGMP, regulating intracellular signal transduction (2). The 11 PDE families contain several variants that show distinct tissue distribution profiles and subcellular localizations (11). PDEs are considered potential drug targets and have been intensively studied using pharmacological approaches (5). Highly selective inhibitors of several PDE families have already been approved for clinical use, or are being evaluated in clinical trials (2). The PDE3 inhibitor milrinone is used to treat acute heart failure. Another PDE3 inhibitor, cilostazol (Pletal^®^), is used to treat ischemic symptoms caused by peripheral arterial occlusive disease and intermittent claudication, and to prevent stroke recurrence (2,6,12). The PDE4 inhibitors cilomilast (Ariflo^®^) and roflumilast (Daxas^®^) are being evaluated clinically for the treatment of chronic obstructive pulmonary disease (2,3,6). Furthermore, the PDE5 inhibitors, sildenafil (Viagra™), vardenafil (Levitra™) and tadalafil (Cialis™), are used for treating erectile dysfunction (2,5,6).

The clinical applications of PDE inhibitors described above were based on the roles of PDEs in normal cells. Meanwhile, PDEs also play pivotal roles in malignant tumor cells. PDE4 is involved in the regulation of apoptosis in chronic lymphocytic leukemia (13). In 2004, we reported that PDE1 is a target molecule of differentiation-inducing factor-1, which inhibits cell proliferation and induces cell differentiation in *Dictyostelium discoideum*, oocytes, mammalian cells and malignant tumor cells (14,15). These findings suggest that PDEs are strongly involved in the differentiation and growth of malignant tumor cells, as well as normal cells, and that PDE signaling may serve as a novel therapeutic target in malignant tumors.

Previously, we examined the expression of PDE1, PDE3 and PDE5 in malignant melanoma cells and demonstrated the involvement of these isoforms in regulating cell proliferation (7–9). In this study, we examined the role of PDE2 in malignant melanoma and demonstrated that only one splice variant, PDE2A2, is expressed in PMP cells. Furthermore, a specific inhibitor of PDE2, EHNA, inhibited the growth of PMP cells in a dose-dependent manner.

PDE2 activity and PDE2A mRNA expression in normal and malignant tumor cells have been reported. PDE2 activity was detected in the cytosolic fraction of normal human umbilical vein endothelial cells (HUVECs), which was isolated by high-performance liquid chromatography (16). PDE2 activity was also detected in malignant tumor cells including mouse malignant melanoma B16 and human glioblastoma A172 cells (17,18). PDE2A mRNA expression was confirmed in the human brain (19), heart (19), vascular endothelial cells (20), and human MG-63 osteosarcoma cells (21), but has not been reported in malignant melanoma cells. PDE2A is present at high levels in the heart, liver, adrenal cortex, brain and epithelial cells (22), although the tissue distribution of individual variants has not yet been reported (4), and differences in kinetic behavior remain unclear. On the other hand, differential subcellular localization of these variants, which may be caused by differences in the N-terminal regions, has been documented (4). PDE2A2 and PDE2A3 are associated with the membrane (4), and mouse PDE2A2 is present in mitochondria (23). In the present study, the expression of PDE2A2 and a non-coding RNA (GenBank NR_026572.2), of which the RT-PCR product was 62 bp longer than that of PDE2A2, was detected in PMP cells. This was confirmed by western blot analysis of PMP cell extracts, which showed an approximately 105 kDa band corresponding to the PDE2A2 protein. The size of this protein is comparable to that (102 kDa) of a protein isolated from HUVECs (24). In addition, we observed that inhibition of PDE2A2 by EHNA affected the proliferation of PMP cells.

Although no abnormalities in the PDE2A gene have been reported in humans, we found four point mutations, one of which (nucleotide position 734) resulted in the substitution of threonine with isoleucine at amino acid 214 in the N-terminal domain (amino acids, 1–233) of PDE2A (GenBank NP_001137311.1), which is adjacent to the GAF-A domain (amino acids, 234–380). Whether this mutation is responsible for the effect of PDE2A2 on cell proliferation is unclear. However, given the influence of the N-terminal regions on the subcellular localization of PDE2A2 variants (4), it is possible that a mutation in the N-terminus altered the subcellular distribution of PDE2A2 in PMP cells. Consequently, intracellular signaling pathways involving PDE2A2, and their effects on proliferation, may be different in normal and PMP cells.

Cell motility is an important factor in the mechanism of metastasis. Although the PDE2 inhibitor, EHNA, suppressed vascular endothelial growth factor (VEGF)-induced HUVEC migration (16), the effect of EHNA treatment on PMP cell migration was negligible, suggesting that PDE2A2 is not involved in the motility of malignant melanoma cells. Further studies will reveal whether these discrepancies can be explained by the changes in subcellular localization of PDE2A2 caused by the mutation in its N-terminal region.

In the present study, EHNA inhibited the proliferation of PMP cells in a dose-dependent manner. The cytotoxic effect of EHNA was assessed using the trypan blue exclusion test. The BrdU cell proliferation assay results showed suppression of DNA synthesis in EHNA-treated PMP cells, and this was thought to be associated with the inhibition of cell growth. Since the PDE2/5 inhibitor, exisulind, triggers apoptosis in human colon carcinoma SW480 cells (25), we investigated a possible similar effect of EHNA on PMP cells. However, the results of TUNEL assays were similar in untreated and EHNA-treated cells, indicating that PDE2A2 is not involved in apoptotic pathways in PMP cells.

**Figure 10:**
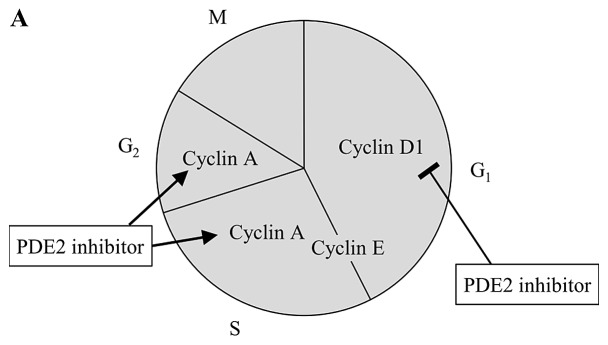

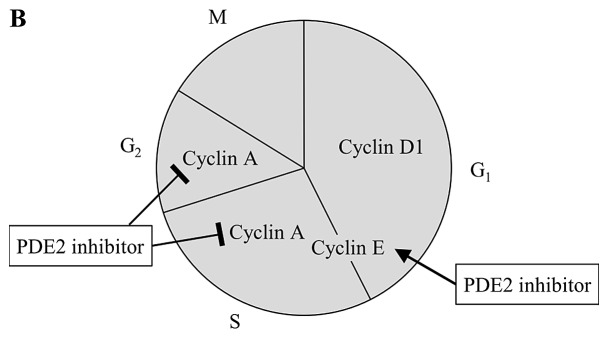
The PDE2 inhibitor plays different roles in HUVECs and PMP cells. (A) In HUVECs, cyclin A was stimulated by the PDE2 inhibitor, whereas cyclin D1 was inhibited. (B) In PMP cells, cyclin A was inhibited by the PDE2 inhibitor, whereas cyclin E was stimulated.

Our findings that EHNA suppressed DNA synthesis, but did not induce apoptosis, in PMP cells suggests a possible effect of the PDE2 inhibitor on cell cycle progression. In HUVECs, EHNA altered EGF-induced cell cycle progression and reduced the proportion of cells in the G_2_/M phase by 19% (16). The present flow cytometric results showed that EHNA treatment increased the proportion of PMP cells in the G_2_/M phase, indicating cell cycle arrest at either the G_2_ or M phase. Given the downregulation of cyclin A and the upregulation of cyclin E by EHNA in PMP cells (Fig. 9), it is possible that the formation of the cyclin A-CDK1 complex was inhibited, causing G_2_-checkpoint arrest and suppression of spindle formation in EHNA-treated PMP cells. Contrary to these changes in PMP cells, upregulation of cyclin A and downregulation of cyclin D1 were previously reported in HUVECs (26). The different effects of EHNA on the cell cycle progression in PMP cells and HUVECs are illustrated in Fig. 10. However, the reasons for these differences are unclear and further studies are necessary. In general, cells are most radiosensitive in the G_2_/M phase (27). In addition, the glutamate release inhibitor riluzole inhibits metabotropic glutamate receptor 1 (GRM1), blocking the MAPK pathway, which is a crucial signaling pathway controlling the pathogenesis of melanoma. Examination of the effects of riluzole in melanoma cells showed an increase in the proportion of cells in the G_2_/M phase and enhanced ionizing radiation-induced cytotoxicity (28). Cyclooxygenase-2 (COX-2) and phosphatidylinositol 3-kinase (PI3K)-AKT signaling are implicated in the radioresistance of melanoma cells, and their respective inhibitors, NS-398 and LY294002, increased the percentage of G_2_/M-arrested cells and decreased clonogenic survival after γ-irradiation of melanoma cells (29). Thus, it is possible that the radiosensitivity of melanoma cells is high in the G_2_/M phase, and if so, a PDE2 inhibitor in combination with radiotherapy may improve the antitumor effect.

In conclusion, this study showed that PDE2A2, which carries an amino acid mutation, is expressed in PMP cells and may downregulate cyclin A, inducing G_2_/M arrest. Thus, the development of drugs targeting PDE2A2 may be useful for the treatment of malignant melanoma.

## Figures and Tables

**Table I:** Primers used to amplify the PDE2A splice variants.

	Sequence
PDE2A1	
Sense primer	5′-GATTGGGGTGTTGAGTCCAG-3′
Antisense primer	5′-TCGATGACAGAGCCCAGACT-3′
PDE2A2	
Sense primer	5′-CACATCCTCATCGCTGTTGT-3′
Antisense primer	5′-AGATGATAGCCTCCCGGACT-3′
PDE2A3	
Sense primer	5′-TGGAAGTGCAGAGCTTAGCC-3′
Antisense primer	5′-GTCGATGACAGAGCCCAGAC-3′

**Table II:** Primers used to amplify the PDE2A2 sequence.

	Sequence
PDE2A2	
Sense primer (Fw1)	5′-GGACCAGGCGAAGCTGTCGC-3′
Antisense primer (Rv1)	5′-CTGAGGCCCAGGAAGGTAGTAC-3′

**Table III:** Primers used to amplify GAPDH, CDKs and cyclins.

	Sequence
GAPDH	
Sense primer	5′-ACAGTCAGCCGCATCTTCTT-3′
Antisense primer	5′-TGAGCTTGACAAAGTGGTCG-3′
CDK1	
Sense primer	5′-GATTCTATCCCTCCTGGTC-3′
Antisense primer	5′-TAGGCTTCCTGGTTTCC-3′
CDK2	
Sense primer	5′-GCTTTCTGCCATTCTCATCG-3′
Antisense primer	5′-GTCCCCAGAGTCCGAAAGAT-3′
CDK4	
Sense primer	5′-ACGGGTGTAAGTGCCATCTG-3′
Antisense primer	5′-TGGTGTCGGTGCCTATGGGA-3′
Cyclin A	
Sense primer	5′-TCCAAGAGGACCAGGAGAATATCA-3′
Antisense primer	5′-TCCTCATGGTAGTCTGGTACTTCA-3′
Cyclin B1	
Sense primer	5′-AAGAGCTTTAAACTTTGGTCTGGG-3′
Antisense primer	5′-CTTTGTAAGTCCTTGATTTACCATG-3′
Cyclin D1	
Sense primer	5′-TGGATGCTGGAGGTCTGCGAGGAA-3′
Antisense primer	5′-GGCTTCGATCTGCTCCTGGCAGGC-3′
Cyclin E	
Sense primer	5′-AGTTCTCGGCTCGCTCCAGGAAGA-3′
Antisense primer	5′-TCTTGTGTCGCCATAATCCGGTCA-3′

## Abbreviations

BrdU: 5-bromo-2′-deoxyuridine
cAMP: adenosine 3′,5′-cyclic monophosphate
cDNA: complementary deoxyribonucleic acid
cGMP: guanosine 3′,5′-cyclic monophosphate
DNA: deoxyribonucleic acid
EHNA: erythro-9-(2-hydroxy-3-nonyl)-adenine
FBS: fetal bovine serum
HUVECs: human umbilical vein endothelial cells
MTS: 3-(4,5-dimethylthiazol-2-yl)-5-(3-carboxymethoxyphenyl)-2-(4-sulfophenyl)-2H-tetrazolium
PBS: phosphate-buffered saline
PDE: phosphodiesterase
RNA: ribonucleic acid
RT-PCR: reverse transcription-polymerase chain reaction
SEM: standard error of the mean
TUNEL: terminal deoxynucleotidyl transferase-mediated dUTP nick end-labeling
VEGF: vascular endothelial growth factor

## References

[1] Thompson JF, Scolyer RA, Kefford RF (2005). Cutaneous melanoma. Lancet.

[2] Beavo JA, Houslay MD, Francis SH, Beavo JA, Francis SH, Houslay MD (2007). Cyclic nucleotide phosphodiesterase superfamily. Cyclic Nucleotide Phosphodiesterases in Health and Disease.

[3] Houslay MD (2009). Underpinning compartmentalised cAMP signalling through targeted cAMP breakdown. Trends Biochem Sci.

[4] Martinez SE, Beavo JA, Francis SH, Houslay MD (2007). PDE2 structure and functions. Cyclic Nucleotide Phosphodiesterases in Health and Disease.

[5] Conti M, Beavo J (2007). Biochemistry and physiology of cyclic nucleotide phosphodiesterases: essential components in cyclic nucleotide signaling. Annu Rev Biochem.

[6] Lugnier C (2006). Cyclic nucleotide phosphodiesterase (PDE) superfamily: a new target for the development of specific therapeutic agents. Pharmacol Ther.

[7] Shimizu K, Murata T, Watanabe Y, Sato C, Morita H, Tagawa T (2009). Characterization of phosphodiesterase 1 in human malignant melanoma cell lines. Anticancer Res.

[8] Murata T, Shimizu K, Narita M, Manganiello VC, Tagawa T (2002). Characterization of phosphodiesterase 3 in human malignant melanoma cell line. Anticancer Res.

[9] Murata T, Shimizu K, Watanabe Y, Morita H, Sekida M, Tagawa T (2010). Expression and role of phosphodiesterase 5 in human malignant melanoma cell line. Anticancer Res.

[10] Kamei T, Inui M, Nakamura S, Okumura K, Goto A, Tagawa T (2003). Interferon-γ and anti-Fas antibody-induced apoptosis in human melanoma cell lines and its relationship to bcl-2 cleavage and bak expression. Melanoma Res.

[11] Omori K, Kotera J (2007). Overview of PDEs and their regulation. Circ Res.

[12] Kambayashi J, Shakur Y, Liu Y, Beavo JA, Francis SH, Houslay MD (2007). Bench to bedside: multiple actions of the PDE3 inhibitor cilostazol. Cyclic Nucleotide Phosphodiesterases in Health and Disease.

[13] Kim DH, Lerner A (1998). Type 4 cyclic adenosine monophosphate phosphodiesterase as a therapeutic target in chronic lymphocytic leukemia. Blood.

[14] Morris HR, Taylor GW, Masento MS, Jermyn KA, Kay RR (1987). Chemical structure of the morphogen differentiation inducing factor from *Dictyostelium discoideum*. Nature.

[15] Shimizu K, Murata T, Tagawa T, Takahashi K, Ishizawa R, Abe Y, Hosaka K, Kubohara Y (2004). Calmodulin-dependent cyclic nucleotide phosphodiesterase (PDE1) is a pharmacological target of differentiation-inducing factor-1, an antitumor agent isolated from *Dictyostelium*. Cancer Res.

[16] Favot L, Keravis T, Holl V, Le Bec A, Lugnier C (2003). VEGF-induced HUVEC migration and proliferation are decreased by PDE2 and PDE4 inhibitors. Thromb Haemost.

[17] Drees M, Zimmermann R, Eisenbrand G (1993). 3′,5′-Cyclic nucleotide phosphodiesterase in tumor cells as potential target for tumor growth inhibition. Cancer Res.

[18] Dunkern TR, Hatzelmann A (2007). Characterization of inhibitors of phosphodiesterase 1C on a human cellular system. FEBS J.

[19] Rosman GJ, Martins TJ, Sonnenburg WK, Beavo JA, Ferguson K, Loughney K (1997). Isolation and characterization of human cDNAs encoding a cGMP-stimulated 3′,5′-cyclic nucleotide phosphodiesterase. Gene.

[20] Seybold J, Thomas D, Witzenrath M, Boral S, Hocke AC, Bürger A, Hatzelmann A, Tenor H, Schudt C, Krüll M, Schütte H, Hippenstiel S, Suttorp N (2005). Tumor necrosis factor-α-dependent expression of phosphodiesterase 2: role in endothelial hyperpermeability. Blood.

[21] Ahlström M, Pekkinen M, Huttunen M, Lamberg-Allardt C (2005). Dexamathasone down-regulates cAMP-phosphodiesterase in human osteosarcoma cells. Biochem Pharmacol.

[22] Gupta R, Kumar G, Kumar RS (2005). An update on cyclic nucleotide phosphodiesterase (PDE) inhibitors: phosphodiesterases and drug selectivity. Methods Find Exp Clin Pharmacol.

[23] Acin-Perez R, Russwurm M, Günnewig K, Gertz M, Zoidl G, Ramos L, Buck J, Levin LR, Rassow J, Manfredi G, Steegborn C (2011). A phosphodiesterase 2A isoform localized to mitochondria regulates respiration. J Biol Chem.

[24] Netherton SJ, Maurice DH (2005). Vascular endothelial cell cyclic nucleotide phosphodiesterases and regulated cell migration: implications in angiogenesis. Mol Pharmacol.

[25] Thompson WJ, Piazza GA, Li H, Liu L, Fetter J, Zhu B, Sperl G, Ahnen D, Pamukcu R (2000). Exisulind induction of apoptosis involves guanosin 3′,5′-cyclic monophosphate phosphodiesterase inhibition, protein kinase G activation, and attenuated β-catenin. Cancer Res.

[26] Favot L, Keravis T, Lugnier C (2004). Modulation of VEGF-induced endothelial cell cycle protein expression through cyclic AMP hydrolysis by PDE2 and PDE4. Thromb Haemost.

[27] Pawlik TM, Keyomarsi K (2004). Role of cell cycle in mediating sensitivity to radiotherapy. Int J Radiat Oncol Biol Phys.

[28] Khan AJ, Wall B, Ahlawat S, Green C, Schiff D, Mehnert JM, Goydos JS, Chen S, Haffty BG (2011). Riluzole enhances ionizing radiation-induced cytotoxicity in human melanoma cells that ectopically express metabotropic glutamate receptor 1 in vitro and in vivo. Clin Cancer Res.

[29] Johnson GE, Ivanov VN, Hei TK (2008). Radiosensitization of melanoma cells through combined inhibition of protein regulations of cell survival. Apoptosis.

